# Hygienic Treatment of Albumenuria

**Published:** 1883-09

**Authors:** George B. Shattuck


THE HYGIENIC TREATMENT OF ALBUMINURIA. BY GEORGE B. SHATTUCK, M.D.

Senator delivered lately a lecture on this subject before the Berlin Medical Society, *which derives its value less from any novelty of the remarks than from the authority of the speaker in regard to renal disorders. Senator's conclusions are confirmatory of the best American and English writers' recommendations as to the treatment of albuminuria. After reminding the reader that the albuminuria is a symptom and measure of a disease, not a disease itself, that the amount of albumen lost—two and a half drachms daily being a very large amount—is not serious, though the local effect of its passage through the renal tissues is very possibly injurious, and alluding to the general recognized inadequacy of drugs in chronic albuminuria, Senator takes up the various points of hygienic treatment which offer hope at least of alleviation and prolonged comparative health, and among such emphasizes especially the following: (1.) The question of the nourishment of patients with nephritis should include a consideration of the influence exercised upon the albuminuria both by the condition of the digestive process itself and by the character of the nourishment. (2.) The rule may be accepted in general that with albuminuria the wants of the system should be supplied rather by frequent small quantities of food than by larger amounts at longer intervals. (3.) Eggs should be forbidden; meat and cheese used sparingly, and of meats preferably veal or poultry; fish is to be recommended; fruit and vegetables are indicated, but the leguminous varieties less so; the use of fat is to be governed by the state of the digestion; spiced, smoked and salted viands are unsuitable; red wine may be used moderately; beer, spirits and the heavier wines are to be avoided; a milk diet is extremely useful, but that it may be sufficiently prolonged bread or some similar addition should be made. (4) Saline or alkaline,—saline waters, warm or cold, according to the case, are found practically to act favorably, and this probably by effect upon the digestion and composition of the blood, as theoretically they should be a renal irritant; saline baths are useful through their congestive and stimulating effect upon the skin. (5) Mus-

* Berlin *Klin Wochschr*, No. 49, 1885.

cular exertion should be very restricted. (6.) An even body temperature should be sought by clothing, by climate, by retirement to bed if necessary. For clothing, flannel should be worn next to the skin ; for a climate a warm and dry one should be selected, free from sudden changes, with a mean temperature from 60° F. to 70° F. (7.) Psychical influences are of great importance in this condition. (8.) With women during menstruation the amount of albumen excreted is always increased, and they should during that period be confined strictly to bed.—*Nashville Journal of Medicine and Surgery.*

———

DR. OSCAR A. KING, Professor of Diseases of the Nervous System in the College of Physicians and Surgeons of Chicago, is about starting a private hospital for the insane at Lake Geneva, Wisconsin. Its location at Lake Geneva, inasmuch as it places the institution beyond the reach of adroic law that govern the commitment of the insane in Illinois, and yet near enough to be taken advantage of by the unfortunates of the State.

THE American Microscopical Society has just closed its session. The meeting was large and exceedingly profitable. At the reception given to the Society by the Calumet Club, of this city, four hundred microscopes were on exhibition, and during the evening at least one change of slide was made, making an exhibition of at least eight hundred unique objects.

WE had the pleasure of a call from our friend, Dr. R. J. Patterson, of Bellevue Place, a hospital for the insane of the private class, at Batavia. His institution is always well filled with patients. It has the entire confidence of those who know anything about its admirable management. It has been in successful operation since 1867.

DR. J. SUYDAM KNOX has returned from a month's vacation, in excellent health.

DR. SIMEON STRAUSSER, of Chicago, is travelling in California.

DR. JOSEPH P. ROSS, of Chicago, is at Cape May.

